# Detection of mycobiota, aflatoxigenic and ochratoxigenic genes, and cytotoxic ability in spices

**DOI:** 10.1002/fsn3.1113

**Published:** 2019-07-08

**Authors:** Eman Gamal Abd Elnaser El‐Dawy, Asmaa Sabry Yassein, Ahmed Hussein El‐Said

**Affiliations:** ^1^ Botany Department, Faculty of Science South Valley University Qena Egypt

**Keywords:** aflatoxins, cytotoxic, genes, ochratoxins, spices

## Abstract

Spices are portions of plants because their properties are used as colorants, preservatives, or medicine. The employments of spices have been known since long time, and the interest in the capability of spices is astounding because of the chemical compounds contained in spices. The molds grow on a variety of different crops and foodstuffs including spices often under warm and humid conditions. The mycobiota of five spice species were surveyed. Forty‐six fungal species were obtained. *Aspergillus flavus* and *A. niger* were the prevalent species recorded. The aflatoxins (AFs) and ochratoxins (OTs) were detected in some samples and isolates. Cumin had the highest concentration of AFs 8.2 ppb, while ginger had a considerable occurrence of OTs 6.7 ppb. *A. flavus* obtained from ginger recorded the maximum concentration of AFs 7.5 ppb, and *A. niger* from turmeric was the highest producer for OTs 3.6 ppb. *omt‐A* and *Aopks* genes were detected in all tested *A*. *flavus* isolates and two out of four *A. niger* isolates. One of the important properties of spices is cancer etiology and prevention. Ginger and sage were the highest cytotoxic against four human tumor cell lines.

## 1 INTRODUCTION

Spices are characterized as any dried, aromatic vegetables or plant materials in the entire, crushed, or ground forms. They incorporate all the parts of herbaceous plants aside from the leaf, which is viewed as an herb (Billing & Sherman, 1998). Spices are utilized in little concentrations for their preservative and medicinal properties (Sethi, Dutta, Gupta, & Gupta, 2013) and also to enhance or change the taste of food (Al‐Mofleh, 2010). In particular, spices have been employed since antiquated circumstances with a view to prevent food spoilage and deterioration and to increase the validity of food (Shan, Cai, Brooks, & Corke, 2007). Investigations have presumed that spices might be extremely helpful since bacteria can develop resistance to conventional anti‐infection agents (Gull et al., 2012). Thus, spices have been utilized as elective drugs particularly among disease patients (Boon, Olatunde, & Zick, 2007). In addition, the herbal medicine has been authenticated to hinder the engendering of tumor cells (Dwivedi, Shrivastava, Hussain, Ganguly, & Bharadwaj, 2011).

*Zingiber officinale* (ginger) is one of the Zingiberaceae family (Bhargava, Dhabhai, Batra, Sharma, & Malhotra, 2012). *Curcuma longa* or “turmeric” as known commonly is vastly utilized as a spice and coloring agent, and it also exhibits stimulating properties (Luthra, Singh, & Chandra, 2001). Cumin seeds (*Cuminum cyminum* L.) belong to the Umbelliferae family and generally implanted in Egypt (Romeilah, Fayed, & Mahmoud, 2010). *Nigella sativa* L. belonging to the Ranunculaceae family is known as dark cumin (Ali & Blunden, 2003). *Salvia officinalis* or sage is a flavoring spice of the Lamiaceae family (Smidling, Mitic‐Culafic, Vukovic‐Gacic, Simic, & Knezevic‐Vukcevic, 2008).

Spices are getting polluted by molds before the drying process during the treatment (Stankovic, Comic, & Kocic, 2006). In addition, the awful states of capacity, ventilation, and high moisture rate are prompt reasons for spoilage of spices by pathogenic microorganisms (Omafuvbe & Kolawole, 2004).

Aflatoxins and ochratoxins A are mycotoxins delivered by few contagious types of the *Aspergillus* and *Penicillium* genera (Frisvad, Thrane, & Samson, 2007). Mycotoxins induce dangerous consequences in living organisms since they exhibit carcinogenic, nephrotoxic, hepatotoxic, and immunotoxic effects (Cao et al., 2013). Aflatoxin and ochratoxin are examined in a variety of dietary products such as spices (Zinedine et al., 2007). Temperature and humidity are considered as the principal factors that control the production of mycotoxin (Griessler, Rodrigues, Handl, & Hofstetter, 2010). The International Agency for Research on Cancer (IARC, 2002) has ordered the naturally occurring blends of aflatoxins as human carcinogens (group 1) and ochratoxin A as potential carcinogen to human (group 2B).

Molecular techniques have been employed as robust methods for detection and identification of fungi (Hassan, Gaber, & El‐Hallous, 2014). Consequently, rapid strategies such as polymerase chain reaction (PCR) have been utilized directly for testing food‐borne pathogens and for confirmation and genotyping of food microbes. The basic feature of PCR is that organisms are not in need to be cultured, at least not for lengthy periods prior to their detection. Moreover, PCR‐based methods that target DNA offer a good way for rapid diagnosis because of their outstanding specificity and sensitivity (Bandyopadhyay et al., 2016), particularly in developing species‐specific primers using multicopy sequences (Awuor et al., 2016).

In the present study, we have evaluated the mycobiota of five types of spices, determination of total AFs and OTs in the most common isolates of *A. flavus* and *A. niger* and also in some samples which contaminated by them, detection of AFs and OTs biosynthesis genes in the same isolates also comparison the genetic diversity by using ISSR‐PCR.

## 2 MATERIALS AND METHODS

### 2.1 Collection of spice samples and mycological isolation

Fifty specimens of spices (10 samples for each) were gathered from different markets in two governorates: Sohag and Qena in Upper Egypt. Each sample was reserved in a sterile polyethylene pack and transferred to the mycological laboratory for fungal examination.

The dilution‐plate technique was applied for the quantitative enumeration of contaminated spices, as mentioned by Christensen (1963). A known weight of tested spices (2 g of each of cumin and black cumin, and 0.3 g of turmeric, sage, and ginger samples) was mixed aseptically in 50 ml of sterile distilled water and shaken vigorously. One ml of the proper dilution was poured in sterilized Petri dishes. Fifteen ml of melted DRBC agar media g/L (peptone, 5; dextrose, 10; KH_2_PO_2_, 1; MgSO_4_·7H_2_O, 0.5; dichloran, 0.002; agar, 15), rose bengal (0.025) and chloramphenicol (0.1) were added as bacteriostatic agents (Baylis, 2003), cooled to 45°C, and poured on the sample suspension in Petri plates which were turned to convey the suspension. Triplicates were read, and the cultures were incubated at 28°C for 7 days. The creating colonies of fungi were isolated, identified, and counted.

### 2.2 Determination of total AFs and OTs producing potential of *Aspergillus flavus* and *A. niger* isolates and also in the samples

The total AFs and OTs producing ability of the isolates were examined by cultivating fungal strains in Czapek yeast extract agar (Ben Fredj, Chebil, & Mlik, 2009) medium for 5 days at 25°C. Total AFs and OTs were extracted by shaking 50 ml of fungal filtrate with methanol (100 ml).

The natural occurrence of total aflatoxins and ochratoxins in five samples (one of each type that highly contaminated) was determined by using a slightly modified method based on the Association of Official Analytic Chemists (AOAC) method (Trucksess et al., 1991). Methanol:water (80:20) solvent (100 ml) and 5 g NaCl were added to 100 g of ground each sample, and the mixture was shacked in a blender at maximum speed for 3 min. Filtration process occurred through fluted filter paper (Whatman 2V; Whatman plc), and the filtrate was diluted (1:4) with water and refiltered through glass‐fiber filter paper. Two milliliters of the glass‐fiber filtrate was placed on an AFs or OTs Test RWB SR Column (VICAM) and allowed to elute at 1–2 drops/s. The columns were washed two times with 5 ml water, and aflatoxin or ochratoxins were eluted from the column with 1 ml high‐performance liquid chromatography (HPLC)‐grade methanol. A bromine developer (1 ml) to the methanol extract was added, and the total AFs or OTs concentrations were read in a recalibrated VICAMSeries‐4 fluorometer set at 360 nm excitation and 450 nm emissions (Lewis et al., 2005).

### 2.3 Molecular detection of AFs and OTs biosynthesis genes

All the molecular steps were employed by Gene analyzer 3121 in Scientific Research Center, Biotechnology and Genetic Engineering Unit, Taif University, KSA.

DNA extraction and purification were performed using DNA Promega Kit DNeasy Blood & Tissue. Two published primer sets were employed for the specific detection of *omt‐A* and *Aopks* genes (Criseo, Racco, & Romeo, 2008). The sequences of primers were as follows: *Aopks*‐F 5′‐CAGACCATCGACACTGCATGC‐3′, *Aopks*‐R 5′‐CTGGCGTTCCAGTACCATGAG‐3′, *omt‐A*F 5′‐ GACCAATACGCCCACACAG‐3′, and *omt‐A*R 5′‐ CTTTGGTAGCTGTTTCTCGC‐3′, respectively. The 549 and 320 bp fragments were amplified, respectively. The used reaction volume in PCR was 25 μl (Hassan et al., 2014). The reactions achieved in a C1000TM Thermo Cycler Bio‐Rad, Germany, involving with denaturation at 94°C for 5 min, followed by 36 cycles at 94°C for 45 s, annealing at 46°C for 45 s, and extension at 72°C for 1 min; then, final step was extended for 7 min at the same temperature (Criseo et al., 2008). The separation of PCR products was accomplished by agarose gel (1.3%) electrophoresis followed by staining with ethidium bromide.

### 2.4 ISSR analysis

Primers utilized in ISSR analysis were obtained from Amersham Pharmacia Biotech. Following the experiments for optimization of component concentrations, PCR amplification of random primers was carried out in 25 μl volume containing 1 μl (20 ng) of genomic DNA, 12.5 μl of Go Taq^®^ Green Master Mix, Promega, USA, 1 μl of primer (20 p.mol), and deionized distilled water (up to a total volume of 25 μl) as described by Hassan et al. (2014). For DNA amplification, the C1000TM Thermo Cycler Bio‐Rad, Germany, was programmed under the conditions involving denaturation at 94°C for 5 min; 40 cycles of denaturation at 94°C for 30 s, primer annealing at 35°C for 1.5 min, primer extension at 72°C for 2.5 min, and final extension step at 72°C for 7 min. Amplified DNA products were analyzed by electrophoresis in 1.5% agarose gel run in TBE. The gels were stained with ethidium bromide (5 μg/ml). 100 bp DNA ladder (Solis Bio‐Dyne^®^) was applied as a standard. DNA was visualized by UV illumination and then photographed by a Bio‐Rad Gel Doc 2000 device.

#### 2.4.1 Data analysis

The amplification results of ISSR‐PCR were recorded for the presence as “1” or absence as “0” and missing data as “9.” The genetic associations between isolates were evaluated by calculating Jaccard's similarity coefficient for pairwise comparisons depending on the proportion of shared bands produced by the primers. The dissimilarity matrix was generated using of neighbor joined technique, and consequently, the dendrogram was reconstructed. The computations were achieved utilizing the NTSYS‐computer program version 2.01 (Rohlf, 2000). For principal component analysis, Jaccard's similarity matrix was amplified.

### 2.5 Cytotoxic effect on human cell lines

All the cytotoxic effect was carried out in Department of Pharmacognosy, National Research Centre El‐Tahrir Str., Dokki, Giza 12622, Egypt.

#### 2.5.1 Preparation of extracts

Ten g of each powdered spice (ginger, cumin, turmeric, sage, and black cumin) was macerated with 50 ml hexane 80% at room temperature for 3 days. The stock solutions were prepared by adding 1ml dimethylsulfoxide (DMSO) to the residue after evaporation. All extracts were stored at 4°C until the cytotoxic test (Esmaeili, Hamzeloo‐Moghadam, Ghaffari, & Mosaddegh, 2014).

#### 2.5.2 Cell lines

The spice extracts were tested against the following cell lines: HCT116 [colon cell line], HePG 2 [human hepatocellular carcinoma cell line], MCF7 [human Caucasian breast adenocarcinoma], and PC3 [prostate cell line].

#### 2.5.3 MTT assay

Cell viability was assessed by the established mitochondrial reduction of yellow MTT (3‐(4,5‐dimethylthiazol‐2‐yl)‐2,5‐diphenyl tetrazolium bromide) to purple formazan. All the succeeding steps were completed under sterilized conditions using a Laminar flow cabinet biosafety class II level (Baker, SG403INT, Sanford, ME, USA). Cells were suspended in RPMI 1640 medium (for HCT116, HePG 2, MCF7, and PC3), 1% antibiotic–antimycotic combination (containing potassium penicillin 10,000 U/ml, streptomycin sulfate 10,000 µg/ml, and amphotericin B 25 µg/ml), and 1% l‐glutamine at 37°C with 5% CO_2_. Cells were batch‐cultured for 10 days and then seeded at a concentration of 10 × 10^3^ cells/well in a fresh complete growth medium in 96‐well microtiter plastic plates at 37°C for 24 hr below 5% CO_2_ using a water jacketed carbon dioxide incubator (Sheldon, TC2323). The media were aspirated, a fresh medium (without serum) was added, and cells were incubated either alone (negative control) or with different concentrations of the sample to give a final concentration of 100–50–25–12.5–6.25–3.125–0.78 and 1.56 ug/ml. After incubation for 48 hr, the medium was re‐aspirated, and 40 ul MTT salt (2.5 μg/ml) was supplemented to each cavity followed by incubation for another four hours at 37°C below 5% CO_2_. For the purpose of dissolving the formed crystals and stopping the reaction, 200 μl of 10% sodium dodecyl sulfate (SDS) in deionized water was supplemented to each cavity and subsequent incubation at 37°C for 24 hr. 100 µg/ml of *Annona cherimolia* extract was used as a positive control that known as natural cytotoxic agent and gives 100% lethality under the same circumstances (El‐Menshawi et al., 2010).

The measurement at the absorbance of 595 nm and at a reference wavelength of 620 nm was performed using the microplate multiwell reader (Bio‐Rad Laboratories Inc., model 3350). The statistical significance between the samples and the negative control (cells with vehicle) was estimated using independent *t* test by SPSS 11 program. DMSO is used for dissolving the plant extracts in final concentrations that do not exceed 0.2% (v/v) in cell assays. The percentage of the change in viability was calculated according to the equation: ((Reading of extract/Reading of negative control) −1) × 100. IC50 and IC90 were calculated by employing a probit analysis using SPSS 11 program.

## 3 RESULTS

### 3.1 Mycobiota contamination of spices

The mycobiota of five kinds of spices marketed in Upper Egypt was investigated in this study. Sage samples were found to be more contaminated than other samples (34,562.46 CFU/g of Sage) as illustrated in Table 1. Studies on the fungal contamination of sage are almost completely absent in the previous literature.

**Table 1:** Fungal counts, frequency of *Aspergilli* on five different spices, total aflatoxins (AFs), and ochratoxins (OTs) in spices

Spice types	Total fungal counts (CFU/g)	*Aspergillus* spp. occurrence (%)	AFs (PPB)	OTs (PPB)
*Cuminum cyminum*	1,933.32	58.19	8.2	4.5
*Curcuma longa*	15,836.52	88.41	2.6	4.1
*Nigella sativa*	4,349.99	85.05	1.9	4.9
*Salvia officinalis*	34,562.46	56.91	6.5	5.4
*Zingiber officinale*	27,838.9	78.24	2	6.7
Total	84,521.19	366.8		

Note

Out of 10 samples from each type of spices, AFs and OTs were determined in the highest contaminant samples .

Eighteen genera comprised of 41 species, and 5 species varieties were obtained from 50 samples of spices that were bought from the markets in Upper Egypt, 2015. The highly isolated fungi in terms of abundance and frequency, which were recovered from the samples of spices, were as follows: *Aspergillus* sp. (71.32% and 100%), *Eurotium chevalieri* (11.76% and 40%), *Emericella nidulans* var. *lata* (11% and 22%), *Penicillium* sp. (4.45% and 66%), and sterile mycelia (1.55% and 34%). The remaining genera were obtained in low or moderate frequencies that formed 9.41% collectively.

### 3.2 Total AFs and OTs producing potentials of *A. flavus* and *A. niger* isolates

Eight isolates of *A. flavus* and *A. niger* isolated from the spice samples were subjected to estimate their AFs and OTs producing potentials. The results are summarized in Table 2. The tested isolates had the abilities to produce total AFs and OTs with the exception of two isolates of *A. niger* from black cumin and sage samples which exhibited no detectable ochratoxin production. The range of AFs produced by *A. flavus* was 2.2–7.5 ppb. The levels of OTs produced by *A. niger* were 3.4 and 3.6 ppb.

**Table 2:** Occurrence, average total counts (calculated per sample), abundance, number of cases of isolation, and frequency of collected species from 50 of different spice samples

Genera and species	Spice types	ATC	Abundance	NCI	*F*%
*Acremonium*	C + S	80.56	0.1	2	4
*A. kiliense*	S	55.56	0.07	1	2
*A. hyalinulum*	C	25	0.03	1	2
*Alternaria*	C + S	525.1	0.62	6	12
*A. alternata*	C + S	516.77	0.61	5	10
*A. fugax*	C	8.33	0.01	1	2
*Aspergillus*	C + Cu + N + S + Z	60,280.53	71.32	50	100
*A. eagypticus*	C	50	0.05	2	4
*A. candidus*	S	166.7	0.28	2	4
*A. clavatus*	*N*	16.67	0.02	1	2
*A. flavipes*	C + N + Z	169.46	0.20	4	8
*A. flavus*	C + Cu + N + S + Z	19,384.06	22.93	39	78
*A. flavus* var*. columnaris*	C	41.67	0.05	1	2
*A. fumigatus*	Cu + N + Z	461.21	0.55	6	12
*A. niger*	C + Cu+ N + S + Z	23,890.4	28.27	50	100
*A. ochraceus*	C + Cu + N + S + Z	4,525.9	5.36	12	24
*A. parasiticus*	S	55.57	0.07	1	2
*A. sydowii*	C + Cu + N + S + Z	5,389.93	6.38	20	40
*A. tamarii*	Z	111.13	0.13	1	2
*A. terreus* var*. africanus*	C + Cu + N + S + Z	875.17	1.04	8	16
*A. terreus* var*. auraus*	C + N + S + Z	2,064.3	2.44	15	30
*A. versicolor*	C + S	2,064.3	2.44	3	6
*Bhusakala olivaceonigra*	S	55.57	0.07	1	2
*Cladosporium oxysporum*	C	8.33	0.01	1	2
*Cochliobolus spicifer*	C	8.33	0.01	1	2
*Emericella nidulans* var*. lata*	C + Cu + S + Z	4,606.47	5.45	11	22
*Epicoccum purpurascens*	C	8.33	0.01	1	2
*Eurotium chevalieri*	C + Cu+ S + Z	9,940.84	11.76	20	40
*Fusarium*	C	33.3	0.04	3	6
*F. oxysporum*	C	8.33	0.01	1	2
*F. solani*	C	25	0.03	3	6
*Mucor hiemalis*	Cu + N+ S + Z	700.13	0.83	7	14
*Paecilomyces carneus*	C	16.67	0.02	1	2
*Papilospora*	Cu + Z	1,333.6	1.58	3	6
*P. immersia*	Z	777.93	0.92	1	2
*P. irregulasa*	Cu + Z	555.67	0.66	2	4
*Penicillium*	C + Cu + N + S+ Z	3,761.7	4.45	33	66
*P. aurantiogriseum*	N + Z	119.46	0.14	3	6
*P. cambertii*	C	8.33	0.01	1	2
*P. chrysogenum*	C + Cu + N + S + Z	2,289.2	2.71	27	54
*P. corylophilum*	S	55.57	0.07	1	2
*P. duclauxii*	C + Cu + S + Z	1,080.77	1.28	8	16
*P. funiculosum*	S	55.57	0.07	1	2
*P. purpurogenum*	C + Cu + N	72.23	0.09	3	6
*P. verruculosum*	C	16.67	0.02	2	4
*P. waksmanii*	C + Cu	63.9	0.08	2	4
*Phoma*	C + N + S	127.79	0.15	3	6
*P. exigua*	C + S	119.46	0.14	2	4
*P. eupyrena*	N	8.33	0.01	1	2
*Rhizopus stolonifer*	N + Z	72.24	0.09	3	6
*Stachybotrys atra* var*. microsporum*	C + S	350.07	0.41	4	8
Sterile Mycelia	C + Cu + N + S + Z	1,308.57	1.55	17	34
*Ulocladium*	Cu + N+ S	1,303.03	1.54	10	20
*U. botrytis*	S	777.93	0.92	4	8
*U. tuberculatum*	Cu + N+ S	525.1	0.62	6	12
Total count		84,521.199			

Abbreviations: C, *Cuminum cyminum*; Cu, *Curcuma longa*; N, *Nigella sativa*; S, *Salvia officinalis*; Z,* Zingiber officinale*.

### 3.3 Natural incidence of total AFs and OTs in spice samples

Five samples out of 50 (which had the highest count of *A. flavus* and *A. niger*) were naturally contaminated with AFs and OTs. The concentration of the AFs in the spice samples ranged from 1.9 to 8.2 ppb with the highest amount found in cumin sample. Likewise, the reading of the OTs ranged from 4.1 to 6.7 ppb, and the highest reading was recorded in ginger sample, as shown in Table 3.

**Table 3:** Frequency of single genes in *Aspergillus flavus*, *A. niger* isolates collected from spice samples

Strain code number	Mycotoxigenic isolate	Source of isolation	Total aflatoxins (ppb)	Aflatoxin gene	Total ochratoxins (ppb)	Ochratoxin gene
31	*Aspergillus flavus*	*Curcuma longa*	2.2	+	0	−
32	*A. flavus*	*Cuminum cyminum*	5.2	+	0	−
33	*A. flavus*	*Zingiber officinale*	7.5	+	0	−
34	*A. flavus*	*Nigella sativa*	2.4	+	0	−
35	*Aspergillus niger*	*Curcuma longa*	0	−	3.6	+
36	*A. niger*	*Cuminum cyminum*	0	−	3.4	+
37	*A. niger*	*Nigella sativa*	0	−	0	−
38	*A. niger*	*Salvia officinalis*	0	−	0	−

Note

+, PCR amplification signal present; −, PCR amplification signal absent.

### 3.4 Molecular characterization of AFs and OTs biosynthesis genes

**Figure 1:**
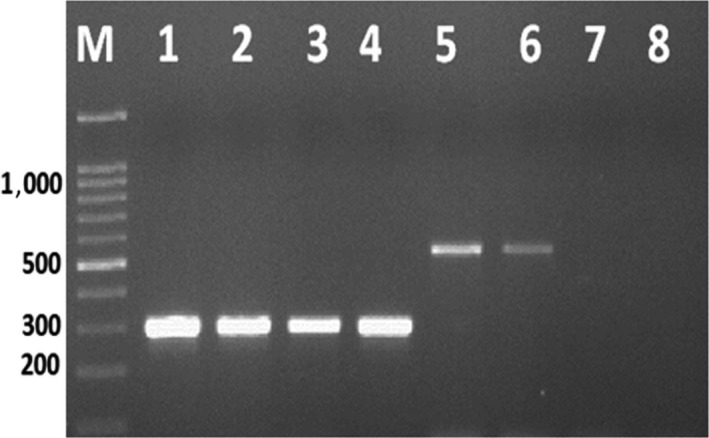
PCR amplification of *omt‐A* and *Aopks* genes (320, 549 bp) for *Aspergillus flavus* and *Aspergillus niger* isolates where 1:4 were *A. flavus* isolates no. 31:34 and 5:8 were *A. niger* isolates no. 35:38

The eight *Aspergillus* isolates identified genes clustered within a 70 kb DNA region in the chromosome that are comprised in the production of AFs and whose DNA sequences have been previously published (Criseo et al., 2008). The polymerase chain reaction (PCR) was applied using two sets of primer for different genes involved in aflatoxin and ochratoxin biosynthetic pathways. The bands of the fragments of *omt‐A and Aopks* genes can be visualized at 320 and 549 bp, respectively (Figure 1). The DNA banding patterns of the examined aflatoxigenic and ochratoxigenic isolates were different. All aflatoxigenic *A*. *flavus* (i.e., isolates, no. 31, 32, 33, and 34) showed DNA fragments that corresponded to the presence of genes. In addition, two of the four *A. niger* isolates (no. 35 and 36) showed the investigated ochratoxin gene.

### 3.5 ISSR‐PCR analysis, genetic distances, and the cluster dendrogram

**Figure 2:**
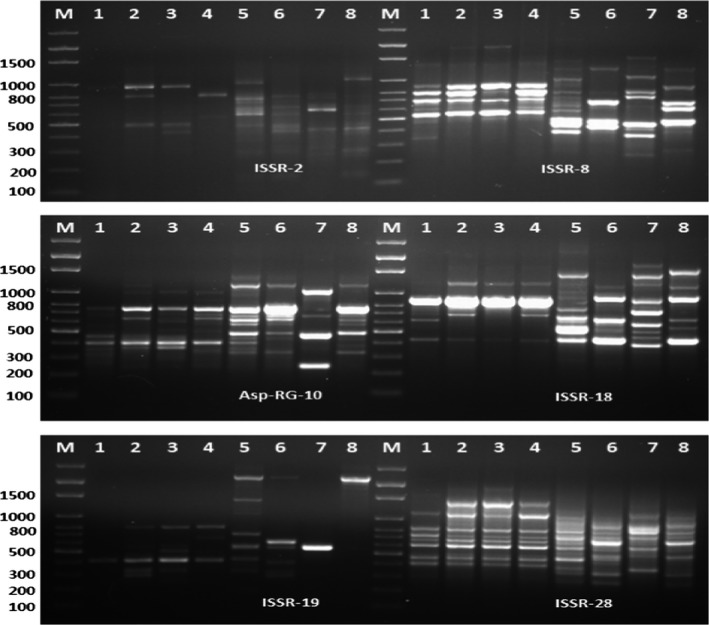
ISSR‐PCR profiles of eight *Aspergillus* isolates generated with 10 primers. M: 100 bp DNA ladder. Whereas, 1 = *Aspergillus flavus‐31*, 2* = Aspergillus flavus‐32*, 3* = Aspergillus flavus‐33*, 4* = Aspergillus flavus‐34*, 5* = Aspergillus niger‐35*, 6* = Aspergillus niger‐36*, 7* = Aspergillus niger‐37*, and 8* = Aspergillus niger‐38*

**Figure 3:**
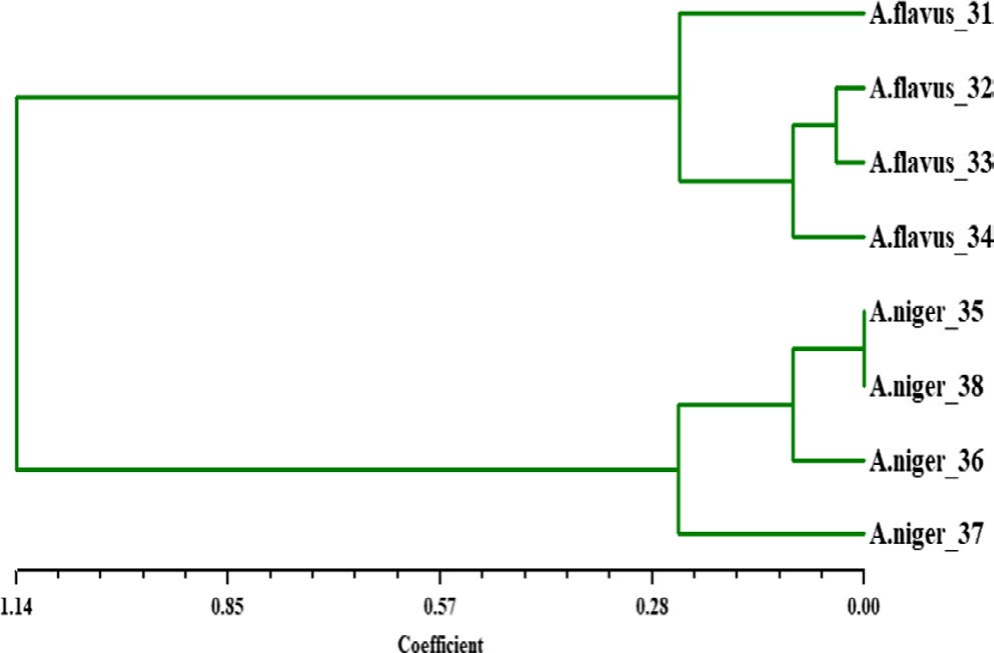
Dendrogram based on 10 ISSR‐PCR primers of eight *Aspergillus* isolates collected from spices

The genomic diversity of *Aspergillus* spp. was investigated by ISSR markers. Results of ISSR‐PCR are illustrated in Figure 3. ISSR‐PCR reactions were completed with eight *Aspergillus* isolates. Six different ISSR‐PCR markers yielded 84 distinct bands of which 57 bands were polymorphic (68%) and only 27 bands were monomorphic (32%). The number of bands for individual ISSR primers varied from 10 bands for ISSR‐16 to 19 bands for ISSR‐28. The highest polymorphism was recorded for ISSR‐19 and the lowest for ISSR‐28. Moreover, the band size of ISSR‐8 ranged from 220 to 2,000 bp (Figure 2).

The genetic similarity and intraspecies differentiation as well as the dendrogram constructed using neighbor‐joint method based on Jaccard's similarity coefficients ranging from 0.00 to 1.14 (Figure 3) were used here. According to the corresponding data, the dendrogram analysis showed that there is genetic distance among native *Aspergillus* sp. They were grouped into two large clusters: The first cluster consisted of *A. flavus*‐31, *A. flavus*‐32, *A. flavus*‐33, and *A. flavus*‐34, and the second main cluster included the four *A. niger* only. The result found that genetic distance among native *Aspergillus* sp. was comparatively low between each species and high within each species in general. The smallest genetic distance (0.00) was estimated between *A. niger*‐35 and *A. niger*‐38 (Figure 3).

### 3.6 Cytotoxic potential of spice extracts

In this experiment, the cytotoxic capability of five extracts of spices against four human tumor cell lines (HCT116, HePG 2, MCF7, and PC3) was probed using the MTT assay (Table 4). The highest cytotoxic action was observed in ginger and sage*.* The crude extract of ginger showed cytotoxic activity against the tested cell lines (IC50: 43.0, 19.8, 43.0, and 18.9  μg/ml, respectively) and (IC90: 77.7, 36.4, 77.7 and 28.8 μg/ml, respectively). The extract of sage had also cytotoxic effects on the four cell lines (IC50: 59.2, 19.07, 59.2, and 22.5 μg/ml, respectively) and (IC90: 95, 36.1, 95, and 34.2 μg/ml, respectively). In contrast, the cytotoxicity of black cumin was found to be considerably low on the four cell lines**.**

**Table 4:** Cytotoxic activity of spices against HCT116, HePG 2, MCF7, and PC3 human tumor cell lines

Scientific Name	IC50 (μg/ml)				IC90 (μg/ml)			
	HCT116	HePG 2	MCF7	PC3	HCT116	HePG 2	MCF7	PC3
*Cuminum cyminum*	N	N	N	24.5	N	N	N	37.6
*Curcuma longa*	45.9	N	45.9	21.7	78.3	N	78.3	34.4
*Nigella sativa*	N	N	N	N	N	N	N	N
*Salvia officinalis*	59.2	19.07	59.2	22.5	95.0	36.1	95.0	34.2
*Zingiber officinale*	43.0	19.8	43.0	18.9	77.7	36.4	77.7	28.8

Abbreviation: N, not effective.

## 4 DISCUSSION

The species *A. flavus* and *A. niger* obtained in this study are described as mycobiota contaminated of spices due to the presence of fungi at preharvest or postharvest depend on storage temperature, seed moisture content, relative humidity, and fungal species. Gherbawy, Shebany, Hussein, and Maghraby (2015) found that crushed chili samples were the preferred mediums for fungal growth among the examined chili products (11.335 × 10^3^ CFU/g of crushed chili). The same results are in line with the returns of Saleem, El‐Said, Moharram, and Abdelnaser (2013); their investigation has disclosed that *Alternaria*, *Aspergillus*, *Emericella*, *Mucor*, *Penicillium*,* Stachybotrys*, and sterile mycelia were the common genera that contaminate anise and cumin seeds. In addition, El‐Gali (2014) has shown that the most prevalent fungal genera isolated from 14 species of spices including turmeric, cumin, and ginger were *Aspergillus *spp.,* Penicillium* spp., *Alternaria *spp., and *Fusarium *spp. Jeswal and Kumar (2015) have found that *Aspergillus flavus* and *A. niger* were the commonest species isolated from 9 kinds of spices also comprising turmeric, cumin, and ginger.

In this study, 100% of tested *A. flavus* isolates were aflatoxigenic and 50% of the tested *A. niger* were ochratoxigenic, and similar results were obtained by Jeswal and Kumar (2015) who reported that all *Aspergillus flavus* isolates from tested spices had the ability to produce aflatoxins. *Aspergillus* species belonging to Circumdati, Nigri, and Flavi sections are the major producers of ochratoxins, whereas aflatoxins are mainly obtained from *Aspergillus*: section Flavi (Rank et al., 2011). Chourasia (1995) found the natural occurrence of aflatoxin B1, citrinin, ochratoxin A, and zearalenone in some herbs such as *Cuminum cyminum* and *Zingiber officinale.* Moreover, Jeswal and Kumar (2015) manifested that AFs amounts were higher than OTA since the ginger, turmeric, and cumin samples had a high percentage of aflatoxins (77.7%, 68.5%, and 64.3%, respectively). The OTA were reported in 55.5% and 57.1% of ginger and turmeric samples, respectively, and the cumin samples were free of OTA. AFB_1_ was detected in 23 samples out of 36 spices (63.9%) in a concentrations range of 0.10 to 26.50 μg/kg (Azzoune et al., 2016).

All aflatoxigenic *A*. *flavus* showed DNA fragments due to the presence of genes, and two of the four *A. niger* isolates showed the ochratoxin gene. Aflatoxigenic aspergilli were detected using PCR based on the intermediated enzymes including the sterigmatocystin O‐methyltransfrase encoding gene *omt‐1* and the regulatory gene *aflR* (Erami et al., 2007). El‐Hamaky, Atef, Yazeed, and Refai (2016) found that a single fragment of about 549 was produced with two positive ochratoxigenic *A. niger* isolates.

The highest cytotoxic activity was observed in ginger and sage extracts. Spices had cytotoxic potential are useful in the development of cancer therapeutics which had increasing importance in the last decade. Nematollahi‐Mahani, Rezazadeh‐Kermani, Mehrabani, and Nakhaee (2007) reported that the ethanol extract of *Teucrium polium* exhibited cytotoxic effects on four cell lines. The IC50 value for each cell line was reported as 90 μg/ml for A‐549, 106 μg/ml for BT‐20, 140 μg/ml for MCF‐7, and 120 μg/ml for PC‐12 cells. Different concentrations of plant extracts (50, 100, and 150 μg/ml) were supplemented to HepG2 cells and were incubated for 48 hr. The experiments revealed that amla, green tea, liquorice, sarpagandha, and periwinkle were cytotoxic to the liver cancer cell line at all varied concentrations. The IC50 values for these were recorded to be less than 150 μg/ml (Rao, Timsina, & Nadumane, 2014). Furthermore, extracts of *Saliva* species especially *S. miltiorrhiza* and *S. officinalis* have been recently documented to possess high antitumor activity in vitro and in vivo (Jiang, Zhang, & Rupasinghe, 2016). Likewise, the ginger extracts have been recorded to manifest antiproliferative actions on many kinds of cancer cells, especially pancreatic cancer cells (Akimoto, Lizuka, Kanemastu, Yoshida, & Takenaga, 2015).

## 5 CONCLUSION

The mycological analysis revealed that sage samples were highly contaminated and that the most prevalent fungi were *Aspergillus* sp. Cumin had the highest reading of AFs, and ginger was the highest for OTs. All the tested isolates of *Aspergillus flavus* and *A. niger* were positive for AFs and OTs production except two isolates of *A. niger* that exhibited no detectable OTs, *omt‐A* gene was found in all *A. flavus* isolates, and *Aopks* genes were detected in 2 out of four isolates of *A. niger*. The most cytotoxic activity was determined in ginger and sage*.* Therefore, our findings reveal that the dryness of the spices after harvesting is the main factor to avoid the development of fungi during storage and open up the possibility that natural compound found in these spices may be used to develop new treatment modality for cancer. However, further investigation is needed to determine the mechanism for its action in carcinoma.

## CONFLICTS OF INTEREST

The authors declare no conflict of interest.

## ETHICAL REVIEW

All experimental protocols and procedures were reviewed and approved by the Department of Pharmacognosy, National Research Centre El‐Tahrir Str., Dokki, Giza 12622, Egypt.

## INFORMED CONSENT

Written informed consent was obtained from all study participants.
